# New clues to the elusive aetiology of nodding syndrome

**DOI:** 10.1093/braincomms/fcad236

**Published:** 2023-09-01

**Authors:** Peter S Spencer

**Affiliations:** Department of Neurology, Oregon Health & Science University, Portland, OR 97239, USA

## Abstract

Nodding syndrome is a paediatric epileptic encephalopathy of unknown aetiology that affects children in impoverished communities of Eastern Africa subject to internal displacement. Set in southcentral South Sudan, where nodding syndrome first surfaced circa 1990, an important new study of recent-onset cases of nodding syndrome examined parasitic, bacterial, viral, immune-mediated, metabolic and nutritional factors associated with the brain disease. Infection with the nematode *Mansonella perstans*, but not with *Onchocerca volvulus*, was the most prominent finding in nodding syndrome cases versus controls. While *M. perstans* is unlikely to be causal of nodding syndrome, investigation of the freshwater habitats, where insect-to-human transmission of the filarial larvae takes place, may reveal a clue as to the aetiology of this neurodegenerative disease. The culpable environmental agent(s) must be able to induce neuroinflammation and tau pathology preferentially in infants and children.

**This scientific commentary refers to ‘Parasitic, bacterial, viral, immune-mediated, metabolic, and nutritional factors associated with nodding syndrome’, by Edridge *et al*. (https://doi.org/10.1093/braincomms/fcad223).**

Edridge *et al.*^1^ report parasitic, bacterial, viral, immune-mediated, metabolic and nutritional factors associated with nodding syndrome affecting subjects within a 20 km radius around Mundri town, Western Equatoria, South Sudan. Nodding syndrome is an idiopathic epileptic brain disorder featured by neuroinflammation and tau pathology; it appears in childhood (3–18 years) with absence and atonic seizures with head nodding, progressing to other seizure types with cognitive deficits and eventually with features reminiscent of parkinsonism. Affected children often have stunted growth and delayed puberty. While the case–control design, the number of nodding syndrome, household and community control subjects and the plethora of advanced analytical techniques are all study strengths, the major contribution is the investigators’ focus on nodding syndrome subjects within 1 year of disease recognition (‘symptom onset’)—presumably head nodding as reported by caregivers. All previous studies of nodding syndrome in Eastern Africa have described longstanding cases where, as the authors note, causative factors may no longer be detectable. Their multiple logistic regression analysis of these recent-onset nodding syndrome cases revealed a very strong association with infection with the nematodes *Mansonella perstans* (MP; OR 7.04) and *Necator americanu*s (OR 2.33) but, notably, no association with *Onchocerca volvulus* (OV).^1^ Since an earlier study^2^ in the same region associated nodding syndrome of unstated duration with a strongly positive serology for both OV (OR 9.2, *P* = 0.00003) and MP (3.22, *P* = 0.05), these new data suggest the former nematode infection is a secondary event unrelated to nodding syndrome aetiology.

Further evidence for this view is the absence of autoimmune antibodies targeting leiomodin-1 resulting from autoantibody cross-reactivity with OV, a hypothesis for the aetiology of nodding syndrome^3^ that has not found recent support.^4,5^ This calls into question efforts elsewhere to prevent nodding syndrome by pesticidal control of OV-transmitting black flies (*Simulium* spp.) and treatment of nodding syndrome patients with doxycycline,^6^ which kills the *Wolbachia* filarial endosymbiont of OV and probably that of MP.^7^ Since polymerase chain reaction evidence for OV^2,4^ and *Wolbachia*^1^ was absent in nodding syndrome CSF, MP and OV may be associated respectively with recent-onset and later cases of nodding syndrome, but it would appear that neither nematode has direct aetiological significance for the paediatric brain disease. The association between *N. americanus* and recent-onset nodding syndrome is also unlikely to be causal of nodding syndrome, but it should be noted that hookworm infection is linked with early cognitive impairment, malnutrition, stunted growth and delayed puberty, features associated with nodding syndrome^2^ and its Nakalanga relative.^8^ Deficiency of plasma zinc (only sodium magnesium and calcium were measured in the present study) may also result in growth retardation and hypogonadism resembling idiopathic hypopituitarism.^9^ In contrast, MP-associated mansonellosis, which is common in North and sub-Saharan Africa, is usually a mild disorder that may include neuropsychiatric disturbances but has no known association with growth defects, delayed puberty or an advancing nodding syndrome–like brain disease.

However, the very strong association found between MP infection and recent-onset nodding syndrome should not be dismissed; indeed, it may be a clue to the real culprit in nodding syndrome. MP is transmitted by biting midges of the genus *Culicoides*, minute haematophagous flies that serve as vectors of nematodes, protozoa and several viruses, particularly arboviruses of domestic livestock.^10^ Preimaginal stages of *Culicoides* spp. thrive in several aquatic habitats, such as streams, lakes, ponds, temporary swamps and saturated soil of the type found in Mundri. This may also be relevant to the stronger seroreactivity and increased odds ratio for malaria in recent-onset nodding syndrome cases^1^ since the transmitting mosquito larvae and pupae live in standing and stagnant water. However, similar water sources are also favoured by snails linked to schistosomiasis, but IgG seropositivity for *Schistosoma* was associated with decreased odds of nodding syndrome.

Although not discussed by Edridge *et al.*,^1^ two of the authors reported previously an association between nodding syndrome in Mundri County and use of river water for drinking, cooking, hand washing and bathing.^11^ Since South Sudan is permeated by rivers, but nodding syndrome is largely confined to parts of Western Equatoria, there must be other factors that drive the selective geographic risk for this childhood brain disease. One important consideration is that, prior to 1990, nodding syndrome was unknown in Mundri County,^2^ a region impacted by episodic civil conflict resulting in population displacement reflected in widely different prevalence rates of nodding syndrome (2.3–13.7%).^2,11^ Civil conflict displaced people to the bush and forced people to rely on all available sources of food and water, including those populated by insects that transmit MP and the malarial parasite. As will be discussed elsewhere, the aquatic habitats favoured by these insects are associated with freshwater cyanobacteria, the secondary metabolites of which are rich sources of bioactive compounds, including vitamins (A, B, C, E and K) and cyanotoxins,^12^ some of which have relevant neurotoxic (notably tauopathic) potential. While water and food sources were not considered by Edridge *et al.*,^1^ they noted that early-stage nodding syndrome was associated with a higher plasma concentration of vitamin E. Their finding of a lower plasma vitamin B12 level correlated with wasting attributed to nodding syndrome–related malnourishment. Increased plasma vitamin A levels were indirectly associated with nodding syndrome, and vitamin B6 levels in CSF were comparable to those in Dutch controls. The importance of poor nutrition in the context of civil conflict and community displacement is illustrated by the Ugandan experience with nodding syndrome, which began among the Acholi in 1995 but had virtually disappeared by 2015 after people had returned from insanitary camps for internally displaced people to their homes. Furthermore, as noted by the authors,^1^ a nutritionally balanced diet (and clean water) combined with anticonvulsive therapy appears to slow progression of the brain disease, another potential clue to nodding syndrome aetiology.

Edridge *et al.*^1^ found no evidence of a CSF viral infection by viral metagenomics, and, strangely, serological profiling by ‘VirScan’ showed that seropositivity to fewer viruses was associated with an increased risk of nodding syndrome. Importantly, no association was found between nodding syndrome and seropositivity for any specific virus. This included measles paramyxovirus, which, while previously found to be negatively associated with nodding syndrome in Mundri County,^1^ was linked by parental self-report to onset of head nodding among Acholi children, and elevated antibody titres were found in a preliminary study of serum of Ugandan patients with nodding syndrome relative to household and community controls.^13^ Measles virus was of potential interest in relation to nodding syndrome because some children with acute infection may, years later, develop subacute sclerosing panencephalitis, a progressive and usually fatal brain disorder also associated with head nodding, neuroinflammation and tau pathology. Rinderpest, the measles-related paramyxovirus, was also of interest in Mundri County because, in 2001, children with nodding syndrome were restricted to ethnic Moru (agropastoralists) and absent from ethnic Dinka people,^1^ who herded cattle subject to rinderpest. Just as cowpox protects against smallpox, one theorized rinderpest might protect against measles and hence prevent a measles-related nodding syndrome. However, since rinderpest was declared eradicated by 2011 and recent-onset nodding syndrome cases lack measles serology,^1^ an aetiological role for a paramyxovirus in nodding syndrome appears to have been ruled out. Indeed, according to Edridge *et al.*,^1^ their ‘exhaustive exploration’ seems to have ruled out a role for any and all known neurotropic viruses in nodding syndrome, which would include human flaviviruses, such as the West Nile virus that was first isolated in northern Uganda. Their screening for known and novel autoimmune antibodies reacting with brain tissue also proved negative.

*Quo vadis*? What aetiologic hypotheses remain for nodding syndrome if neurotropic viruses, parasites, bacteria and autoimmune factors are ruled out? Since community displacement is geographically and temporally associated with nodding syndrome in South Sudan and Uganda, it will be important to develop a detailed understanding of the changes in environmental exposures that result from displacement, whether biological or chemical in nature. Therein may lie the key to the aetiology, prevention and treatment of this mysterious disorder. The central question is what environment agent might trigger tau pathology in the brain of infants and children, because any theory will be incomplete if it fails to explain this key neuropathological feature of nodding syndrome. Further, it will not be lost on readers of this journal that the discovery of an environmental agent that promotes tau pathology in the human brain may have relevance to the aetiology of certain other neurodegenerative disorders worldwide.
